# Evaluation of an Electronic Nose Coupled with In Vitro Fecal Fermentation as a Screening Tool for Fecal Odor in Cats

**DOI:** 10.3390/ani16050801

**Published:** 2026-03-04

**Authors:** Koramit Jenjirawatn, Attawit Kovitvadhi, Songyos Chotchutima, Pipatpong Chundang, Sathita Areerat, Kunaporn Homyog, Nattaphong Akrimajirachoote

**Affiliations:** 1 Animal Health and Biomedical Science Program, Faculty of Veterinary Medicine, Kasetsart University, Bangkok 10900, Thailand; koramit.jen@mahidol.ac.th; 2 ANLAR Service Co., Ltd., Pathum Thani 12130, Thailand; attawitthai@gmail.com; 3 Department of Agronomy, Faculty of Agriculture, Kasetsart University, Bangkok 10900, Thailand; fagrsyc@ku.ac.th; 4 Department of Physiology, Faculty of Veterinary Medicine, Kasetsart University, Bangkok 10900, Thailand; pichandang@gmail.com (P.C.); sathitameen@gmail.com (S.A.); 5 Center of Veterinary Diagnosis, Faculty of Veterinary Science, Mahidol University, Nakhon Pathom 73170, Thailand; kunaporn.hom@mahidol.ac.th

**Keywords:** dietary fecal deodorant, fecal inoculation, electronic nose, feline diet

## Abstract

In vitro fecal inoculation coupled with gas chromatography–mass spectrometry (GC-MS) has been used for evaluating fecal deodorants. However, high cost and complex data interpretation limit its routine application. An electronic nose (eNose) offers a rapid, cost-effective alternative. This study aimed to evaluate the eNose as a screening tool for fecal odor compared with solid-phase microextraction gas chromatography–mass spectrometry (SPME GC-MS) and to examine the in vitro effects of fecal deodorant supplements on fecal odor profiles. Feces from ten healthy cats were serially diluted (1:1 to 1:8) and analyzed using both instruments. Four dietary supplements—*Yucca schidigera* extract (YSE), *Quillaja saponaria* extract (QSE), fructooligosaccharides (FOS), and oat beta-glucans (OBG)—were tested at concentrations of 0.0, 0.2, 0.4, and 0.8 g/100 mL. The eNose showed comparable performance to GC-MS in discriminating among sample dilutions. In vitro fermentation showed that FOS and OBG significantly increased volatile fatty acid (VFA)-related sensor responses while signals linked to ammonia and sulfur compounds were reduced. QSE had minimal effect, whereas YSE produced moderate changes. The total sensor response intensities did not differ between treatments. These findings indicate that prebiotic supplements exert stronger effects than saponin-based supplements and highlight the potential of eNoses with in vitro fermentation for rapid screening of fecal deodorants.

## Simple Summary

Evaluating the effectiveness of dietary fecal deodorizing supplements in cats is time-consuming and costly when conventional analytical methods are used. Previous studies have relied on in vitro fecal inoculation combined with gas chromatography–mass spectrometry, but data interpretation is complex and limits its use as a rapid screening tool. Electronic nose (eNose) technology offers a faster and more practical alternative. This study evaluated an in vitro fecal inoculation system coupled with an eNose as a rapid screening method for dietary fecal odor modulation in cats. Using this approach, four commonly used supplements—*Yucca schidigera* extract, *Quillaja saponaria* extract, fructooligosaccharides, and oat beta-glucans—were assessed. The method effectively differentiated odor profile changes induced by dietary supplements. Among the tested ingredients, *Quillaja saponaria* extract showed the weakest odor-modulating effect. *Yucca schidigera* extract produced minor alterations in odor characteristics, whereas fructooligosaccharides and oat beta-glucans demonstrated greater odor-modulating effects relative to the control. These findings support the use of an eNose-based in vitro system as a practical screening tool for fecal deodorant efficacy in feline nutrition research.

## 1. Introduction

Pet parenting has become a modern societal trend, with many pet owners perceiving their pets as children or close companions and allowing them access to private living areas [1]. One emerging problem associated with this pet-ownership style is fecal odor. Fecal odor originates from various volatile compounds, including volatile fatty acids (VFAs), branched-chain volatile fatty acids (BCVFAs), ammonia, sulfur-containing compounds, indoles, and phenols. In cats, VFAs, phenolic compounds, and indoles are suspected to be the main contributors to malodor [2]. These compounds are byproducts of the microbial fermentation of undigested nutrients and can be reduced through appropriate nutritional management [3].

Researchers have focused on dietary protein and fermentable carbohydrate concentrations as key nutritional strategies for reducing fecal malodor in animals. Higher dietary protein levels are associated with increased emissions of ammonia, BCVFA, *p*-cresol, indoles, and skatole. Protein source also plays a role; previous studies have shown that animal-based proteins tend to produce more malodorous feces compared to plant-based or hydrolyzed proteins [4]. In addition, fermentable carbohydrates can decrease malodorous compounds while increasing VFA production, which can contribute to an odor with a different profile [5].

To mitigate fecal odor, various dietary fecal deodorant (DFD) supplements designed to reduce malodorous gas emissions have been incorporated into pet diets. *Yucca schidigera* is commonly used in feline diets due to its saponin content, which can help reduce odor by binding to ammonia and hydrogen sulfide, altering gut microbiota, and inhibiting urease activity [6,7,8]. *Quillaja saponaria*, another saponin-containing plant, has also been utilized in livestock diets [9]. Fermentable fibers, such as fructooligosaccharides and beta-glucans, although not primarily developed for odor control, can modulate the gut microbiota and reduce the production of malodorous compounds in animal feces [10,11]. However, the use of individual control groups in each study limits direct comparisons and makes it difficult to assess the relative efficacy of these supplements.

Currently, three primary methods are used to assess odors: human sensory panels, gas chromatography–mass spectrometry (GC-MS), and electronic nose (eNose) systems. Human sensory panels rely on scoring by expert panelists or trained volunteers [3]. Although this is the only method that directly reflects human perception of smell, it is inherently subjective, expensive, and especially challenging when evaluating malodors. GC-MS and eNose systems both measure the volatile organic compounds (VOCs) present in samples, but neither directly represents human olfactory perception.

GC-MS precisely quantifies individual VOCs [2] but requires substantial investment in equipment and is relatively time- and cost-intensive per sample [12]. In contrast, the eNose, though less specific due to its limited sensor array, can detect and discriminate odor patterns and relative signal intensities [13], enabling rapid and cost-effective screening analyses. Although both GC–MS and eNose technologies have been widely applied in odor assessment, the integration of eNose analysis with a feline in vitro fecal fermentation system for the comparative evaluation of dietary deodorant supplements has not been systematically evaluated. The present study does not introduce a new detection technology but contributes to the field by demonstrating the feasibility of combining eNose analysis with an in vitro batch fecal inoculation model as a practical screening platform for feline fecal odor modulation. Therefore, this study aimed to evaluate the eNose as a screening method for fecal odor compared with SPME GC-MS and to examine the in vitro odor-modulating effects of common fecal deodorant supplements, namely *Yucca schidigera* extract (YSE), *Quillaja saponaria* extract (QSE), fructooligosaccharides (FOS), and oat beta-glucans (OBG).

## 2. Materials and Methods

### 2.1. Animals

A total of ten clinically healthy cats, aged 1 to 7 years, were included in this study. Each cat was individually housed in a separate cage. Body weight, body condition score, and fecal score were assessed, and regular physical examinations were performed to confirm that all cats remained clinically healthy. All cats were fed a complete and balanced commercial kibble diet (Table 1) with ad libitum access to water. This study was approved by the Kasetsart University-Institute Animal Care and Use Committee (KU-IACUC), under approval letter ID No. ACKU67-VET-015.

### 2.2. SPME GC-MS

A solid-phase microextraction (SPME) GC-MS apparatus (7890A/5975C GC-MS, Agilent Technologies, Santa Clara, CA, USA) was used to identify the VOCs associated with fecal odor. Approximately 1 g of each sample was weighed into 10 mL glass vials with leak-proof sealed caps. The vials were incubated at 39 °C for 10 min, followed by 50 min of volatile compound extraction using a DVB/Carbon WR/PDMS fiber (Supelco, Sigma-Aldrich, Bellefonte, PA, USA). After extraction, the SPME fiber was thermally desorbed in the GC injector at 240 °C for 5 min prior to analysis. The GC was performed on a 30 m × 0.25 mm × 0.25 µm HP-5ms column (Agilent J&W GC Columns, Agilent Technologies, Santa Clara, CA, USA). The inlet temperature was set at 240 °C. The column temperature was initially held at 40 °C for 3 min, then increased at 7 °C per minute until reaching 240 °C, where it was held for 8 min, used helium as carrier gas, and carrier gas flow rate was set at 1 mL/minute. Mass spectrometry was operated in scan mode with the following settings: analyzer temperature at 150 °C, ion source temperature at 230 °C, and a solvent cut time of 0.5 min. Scanning was conducted over a mass range of 34 to 350 Daltons. Compound identification was performed using the NIST 17 mass spectral library, with a similarity index threshold of ≥60%.

### 2.3. eNose

For fecal odor determination using the eNose (MUI nose 1.1, MUI Robotics, Nonthaburi, Thailand), approximately 1 g of each sample was weighed, transferred into 100 mL Duran bottles, and stored at −20 °C until analysis. Prior to measurement, samples were incubated at 39 °C for 30 min. An array of eight sensors for detecting malodor (Table S1), as previously described [14], was selected to evaluate fecal odor intensity and characteristics. The selected sensor array is designed to detect broad classes of malodorous gases, including sulfur-containing compounds, nitrogenous compounds, and organic acids, which are commonly generated during fecal fermentation across animal species. Although the relative abundance of these compounds may differ in obligate carnivores such as cats, the principal volatile classes remain conserved.

Measurements were conducted according to the VDI/VDE 3518 guideline for odor-related measurements using an electronic nose. The reference time was set to 3 min, the sampling time to 2 min, and the gas flow rate to 1 L/min.

The sensor response percentage (*S*) was calculated using the following equation:

$$S=\frac{R_{f}-R_{s}}{R_{s}}\times 100$$

where

*S* = electronic nose sensor response percentage.

*R_f_* = sensor resistance of reference gas (air zero).

*R_s_* = sensor resistance of sample odor.

### 2.4. Experiment 1: Evaluation of eNose Efficacy Compared with SPME GC-MS

To evaluate the potential of the eNose device for fecal odor assessment, SPME GC-MS was used as a comparison method. Baseline sensor responses were recorded and are presented in Supplementary Table S2 and Figure S1. A representative response profile of sensor S1 is shown in Figure S2. In brief, the baseline resistance of the sensor array was monitored over a two-day period under controlled laboratory conditions to assess sensor stability and drift. Stability was quantified using the relative standard deviation (%RSD) across repeated measurements (Table S2) Sensors S_1_, S_3_, S_4_, S_5_, S_6_, and S_8_ demonstrated excellent baseline stability, with %RSD values ranging from 0.59% to 5.19%. Sensors S_2_ and S_7_ exhibited higher baseline variability (%RSD > 16%), which is consistent with the known sensitivity of metal oxide semiconductor (MOS) sensors to environmental fluctuations and aging effects [15]. To minimize the influence of inter-day baseline drift, sensor responses were expressed as normalized relative changes (percentage response) rather than raw resistance values. This normalization procedure reduces the impact of baseline shifts and allows consistent comparison of response magnitudes across measurement days [16]. Although S_2_ and S_7_ showed higher baseline variability, they provide complementary selectivity profiles. Therefore, they were retained in the sensor array to preserve overall chemical discrimination capacity.

Fresh fecal samples were collected from ten clinically healthy adult cats, homogenized, and stored in screw-cap plastic tubes at −20 °C. Prior to analysis, each sample was diluted with distilled water at ratios of 1:1, 1:2, 1:4, and 1:8. Aliquots (1 mL) of each dilution were transferred into glass vials with leak-proof sealed caps for SPME GC-MS analysis, and into 100 mL Duran bottles for eNose analysis. All SPME GC–MS and eNose measurements were performed in technical duplicate for each sample dilution.

### 2.5. Experiment 2: Evaluation of Odor-Modulating Effects of Dietary Supplements Using In Vitro Fermentation Combined with eNose Analysis

A batch fecal inoculation system was employed to determine the effect of DFD. The composition of the pre-reduced anaerobically sterilized (PRAS) medium, modified from previous studies [17], is shown in Table 2. A dry undigested fraction, prepared using a two-step in vitro digestion method [18] from a complete and balanced feline diet, was used as the nutrient source in the medium [19]. The proximate compositions of the diet and the undigested fraction used in the PRAS medium are presented in Table 1. Four DFD supplements, YSE, FOS, QSE, and OBG, were evaluated. Each supplement was mixed into PRAS medium at concentrations of 0.2, 0.4, and 0.8 g/100 mL, and a control group (CON) without supplements was included for comparison.

For inoculation, fresh fecal samples from three healthy cats used in Experiment 1 were randomly collected, pooled, homogenized, and suspended at a 1:10 (*w*/*v*) ratio in pre-warmed PRAS medium. The fecal inoculum was then distributed to plastic tubes, each containing 400 µL of fecal inoculum and 3600 µL of PRAS medium, with or without deodorant supplements. Inoculations were incubated at 37 °C under anaerobic conditions with shaking at 370 rpm for 24 h. After fermentation, 1 mL aliquots of the final fermentation product were transferred to 100 mL Duran bottles and stored at −20 °C until odor determination was performed using the eNose. Each inoculation was performed in duplicate.

The methodology described in this study is intended as a screening approach for dietary fecal deodorant supplements in cats. It facilitates relative comparisons of odor-modulating effects among treatments under in vitro conditions and does not provide calibrated quantification of perceived odor intensity or direct representation of human olfactory perception.

### 2.6. Statistical Analysis

The continuous variables are presented as arithmetic means and standard deviation, or as standard error of the mean when inferential statistics are performed. For all continuous variables, the parametric assumptions were assessed: normality using the Shapiro–Wilk test and homogeneity of variance using Levene’s test. Hierarchical cluster analysis (HCA) with Ward’s linkage and principal component analysis (PCA) were used to assess the clustering efficacy between the eNose and SPME GC-MS. In Experiment 2, the fermentation vial was considered the experimental unit for statistical analysis, as pooled fecal samples from three donor cats were used to prepare a composite inoculum prior to treatment allocation. Each treatment–concentration combination was tested in duplicate (technical replicates). Mean differences in sensor responses between the treatment groups were analyzed using two-way ANOVA, with treatment and concentration as fixed factors, followed by Tukey’s test or Fisher’s LSD test for post hoc comparisons. Orthogonal polynomial contrasts were used to determine the linear and quadratic effects of concentration. A heatmap combined with HCA was also applied to the sensor responses to the final fermentation products for visualization. Statistical significance was set at *p* < 0.05. All analyses were performed using SPSS v. 30 (IBM corp., Armonk, New York, NY, USA) and RStudio software v. 2023.09.1+494 (Posit Software, Boston, MA, USA).

## 3. Results

### 3.1. Experiment 1: Evaluation of eNose Efficacy Compared with SPME GC-MS

**Figure 1:**
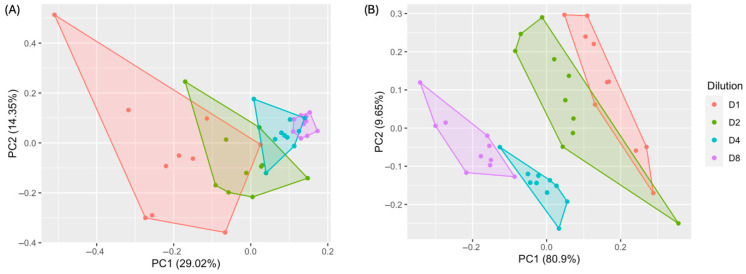
PCA plots of fecal odor results from the same fecal samples (*n* = 10) obtained using (**A**) SPME GC-MS and (**B**) eNose. Colors indicate sample dilution levels: D1, 1:1; D2, 1:2; D4, 1:4; D8, 1:8.

The VOC peak areas from the SPME GC-MS data and the sensor response percentages from the eNose for each fecal dilution from the ten cats are presented in Table 3 and Table 4, respectively. To visualize and compare the odor characteristics across methods, PCA was performed. The PCA of the SPME GC-MS data (Figure 1A) explained 29.02% and 14.35% of the total variance of PC1 and PC2, respectively. In contrast, the PCA of the eNose results (Figure 1B) explained 80.9% of the total variance for PC1 and 9.65% for PC2. These findings indicate that the eNose showed improved visual separation of dilution groups in PCA.

**Figure 2:**
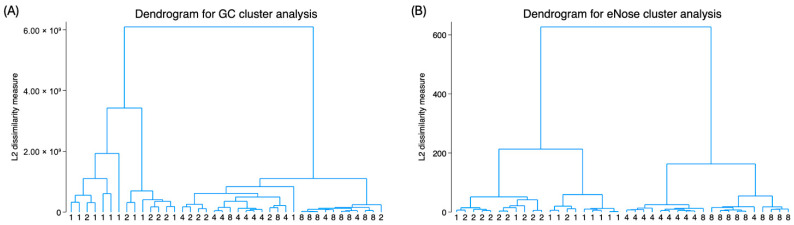
Hierarchical cluster analysis using Ward’s method and dendrograms of (**A**) SPME GC-MS and (**B**) eNose results. Numbers at bottom indicate the dilution factor of each sample.

To further compare the clustering efficiency between the SPME GC-MS and the eNose data, HCA with Ward’s linkage was applied, and the resulting dendrograms are shown in Figure 2. The discrimination between the 1:1 and 1:2 dilutions was unclear for both detection methods. However, the HCA results showed that the eNose effectively clustered samples at 1:4 and 1:8 dilutions, whereas SPME GC-MS failed to clearly distinguish between these dilutions.

### 3.2. Experiment 2: Evaluation of Odor-Modulating Effects of Dietary Supplements Using In Vitro Fermentation Combined with eNose

**Figure 3:**
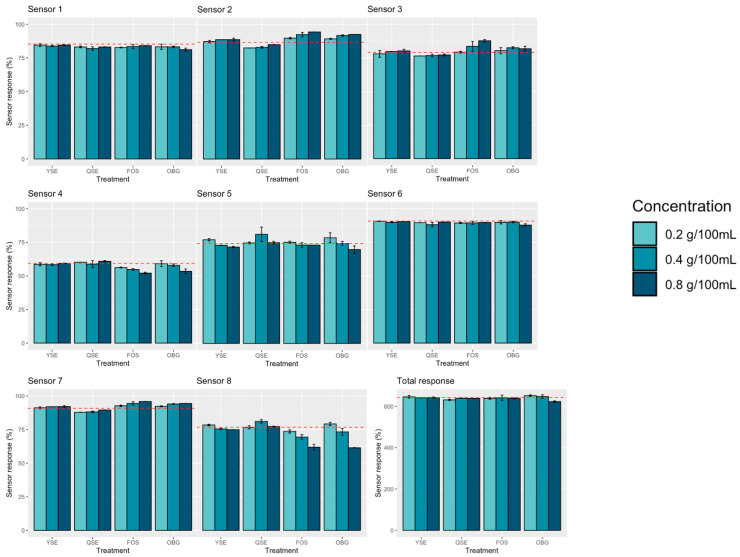
Bar graphs of eNose sensor responses to final in vitro fermentation products at each treatment concentration. Red dashed line indicates response of control. CON, control; YSE, *Yucca shidigera* extract; QSE, *Quilaja saponaria* extract; FOS, fructooligosaccharide; OBG, beta-glucan. Colors represent concentrations of in vitro inoculation: 0.2, 0.4, and 0.8 g/100 mL.

The eNose sensor responses following in vitro fermentation are summarized in Table 5 and illustrated in Figure 3. Overall, supplementation primarily altered the distribution of individual sensor responses rather than total sensor intensity.

Significant treatment effects were observed for sensors S2, S3, and S7 (*p* < 0.05). For S2 and S7, responses increased at 0.4 and 0.8 g/100 mL compared with 0.2 g/100 mL, with higher concentrations differing significantly from the control. For S3, responses were higher in the FOS and OBG groups compared with YSE and QSE, although no significant differences from the control were detected. These results reflect concentration-dependent changes in sensor response patterns.

Sensors S4 and S8, which are responsive to ammonia- and sulfur-associated volatile signals, showed lower responses in the FOS and OBG groups compared with YSE and QSE. For S4, a significant interaction effect was observed, with responses in the FOS and OBG groups decreasing as concentration increased, whereas YSE and QSE remained comparable to the control. For S8, responses generally decreased with increasing concentration in the FOS, OBG, and YSE groups, while QSE showed a non-linear response pattern.

Sensors S1, S5, and S6 showed limited differences among supplement groups. Although S1 and S6 exhibited lower responses compared with the control at certain concentrations, no consistent dose-dependent pattern was observed across treatments.

For the total sensor response, a significant interaction effect was observed (*p* < 0.05). Although total responses were comparable among treatments at each concentration, a concentration-dependent decline was observed, with the lowest response at 0.8 g/100 mL. The OBG group showed a gradual decrease with increasing concentration. Linear polynomial trends were observed for total response and all individual sensors except S5 (*p* < 0.05), whereas a quadratic response was detected only for S8 (*p* < 0.05).

**Figure 4:**
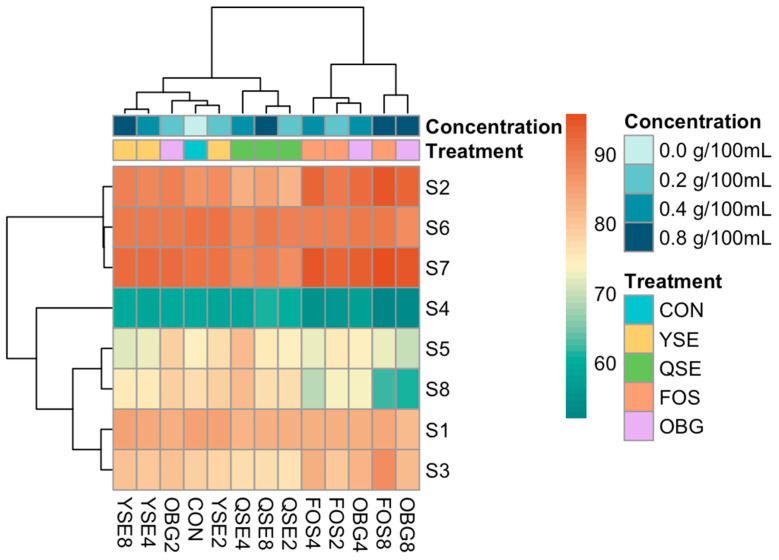
Heatmap and hierarchical cluster analysis dendrogram of in vitro end fermentation product odor results from eNose using Ward’s method. CON, control; YSE, *Yucca shidigera* extract; QSE, *Quilaja saponaria* extract; FOS, fructo oligosaccharide; OBG, beta-glucan. Number behind each treatment indicates concentration of in vitro inoculation: 2, 4, and 8 refer to 0.2, 0.4, and 0.8 g/100 mL, respectively.

The heatmap and HCA diagram of the in vitro end fermentation odor analysis using Ward’s linkage are shown in Figure 4. Four distinct clusters were observed. At the highest concentration (0.8 g/100 mL), FOS and OBG were grouped together. Additionally, FOS at 0.2 and 0.4 g/100 mL clustered with OBG at 0.4 g/100 mL. The odor profile of OBG at 0.2 g/100 mL grouped with the CON and YSE groups for all concentrations. In contrast, QSE formed a separate cluster at every concentration, indicating a distinct odor profile.

## 4. Discussion

The present study demonstrates the feasibility of integrating eNose analysis with an in vitro batch fecal inoculation system as a practical screening platform for evaluating feline fecal odor modulation. The eight-sensor eNose array in this study served as an effective analytical tool for in vitro assessment of fecal odor in cats and successfully discriminated fecal odor patterns across dilution levels, supporting its potential as a rapid screening alternative to SPME GC–MS. The combined eNose and in vitro fermentation approach required smaller diet sample quantities, fewer animals, shorter experimental durations, and no human sensory panelists, making it an efficient and practical screening method for evaluating DFD. With appropriate sensor configurations, this system may also be applicable for assessing fermentation-related volatile signals or microbially derived metabolites associated with prebiotics, probiotics, and other bioactive dietary components. In comparing the fecal odor-modulating effects of the tested supplements, the prebiotics FOS and OBG exhibited more pronounced shifts in sensor response patterns than saponin-containing plant extracts YSE and QSE. These findings suggest the potential of FOS and OBG as candidate supplements for fecal odor control in cats.

The SPME GC–MS results in this study identified a variety of malodorous VOCs, consistent with previous reports [2,3,20,21]. However, several VOCs reported in earlier studies were not detected in the present study, possibly due to differences in mass spectrometer setting. In this study, the major VOCs identified in cat feces included *p*-cresol, VFAs, indole, benzaldehyde, silanediol, and phenol. The eNose sensors were specifically chosen to detect malodors originating from animal feces [14] and demonstrated satisfactory discrimination performance among different sample dilutions compared with SPME GC-MS.

To compare fecal odor among samples with similar odor profiles, the total sensor response may serve as an approximate indicator of odor intensity. However, interpretation becomes more complex when samples exhibit differing odor profiles. In the present study, although the total sensor response did not change significantly, fermentation in the prebiotic groups was associated with a shift in sensor response patterns from sulfur-related signals toward VFA-related signals, consistent with a previous report [3]. This compositional shift may influence human perception of feline fecal odor. Previous research has shown that fecal odor scores assessed by human panelists correlate strongly with hydrogen sulfide concentration [22]. Therefore, higher responses from sulfide-related sensors may indirectly indicate more unpleasant odor perception by humans than those from VFA-related sensors, even when total sensor responses are similar.

YSE has been used as a DFD in cats [23,24]. However, its efficacy as a dietary supplement for fecal odor control remains controversial, particularly in high-protein diets. Previous studies employing human sensory panels have reported a non-linear response to YSE supplementation. Specifically, a quadratic effect was observed for cats, with a medium dose (250 ppm) increasing fecal malodor compared with the control [25]. Similarly, in dogs, fecal odor reduction was observed at a medium dose (500 ppm), whereas both lower (250 ppm) and higher (750 ppm) doses increased malodor when assessed by human panelists [26]. In the present study, although human sensory perception was not evaluated, YSE supplementation showed a linear reduction in ammonia-related sensor responses, accompanied by increased signals associated with VFAs and a slight increase in sulfur-related compounds. These findings suggest that YSE may modulate specific gaseous components of fecal odor rather than the overall odor. Therefore, the apparent discrepancy between instrumental measurements and reported human sensory outcomes may reflect differences in odor characteristics rather than changes in the concentration of individual volatile compounds, highlighting an inherent limitation of eNose-based analysis, which cannot directly mimic human olfactory perception.

*Quillaja saponaria*, another saponin-containing plant [27], has recently been used to reduce fecal odor in livestock [9]. In the present study, at all tested concentrations, QSE did not significantly reduce the eNose sensor responses associated with malodor. This was particularly evident in sensors 4 and 6, which detect sulfur-containing organic compounds and methane, respectively. These findings are consistent with previous studies reporting that QSE is effective in reducing ammonia, but not other malodorous VOCs [28,29,30]. In addition, QSE supplementation has been shown to be less effective than FOS in reducing noxious gas emissions in growing pigs [31]. In this study, in vitro fermentation with QSE showed lower responses by sensors 2, 3, and 7, which are associated with VFAs, compared with FOS and OBG.

Furthermore, QSE supplementation did not reduce the responses of sensors 5 and 8, which are associated with ammonia detection. In contrast, YSE supplementation showed a decreasing trend in ammonia-related sensor responses as concentration increased, despite having a similar proposed saponin-mediated mechanism of action. This finding is consistent with a previous study in ruminants, which suggested that differences in glycofraction and saponin concentration between YSE and QSE may contribute to their differential effects [32].

For the prebiotic groups (FOS and OBG), FOS has been widely used to modulate the gut microbiome [33,34] but is generally not supplemented for the primary purpose of fecal deodorant. In this study, fermentation of FOS increased responses from sensors 2, 3, and 7 (VFAs) and decreased responses from sensors 4 and 8 (sulfur-containing compounds and ammonia, respectively). These findings align with previous studies demonstrating that FOS supplementation in cats reduces sulfur compounds while enhancing VFA production [35,36]. Beta-glucans exhibit diverse conformations depending on their source, leading to differences in branching structure, chain length, and molecular weight that influence their fermentation by the gut microbiota [37]. Among the different types, beta-1,3/1,4-glucans derived from oats and barley have demonstrated stronger prebiotic activity [38].

In the present study, microbial fermentation of OBG showed a trend similar to that of FOS, with increased responses from sensors associated with VFAs and decreased responses from sensors detecting ammonia and sulfur-containing compounds, while total sensor responses remained unchanged. These findings are consistent with previous studies in which in vitro supplementation of OBG in rats [39] and pigs [40] increased total VFA levels and reduced ammonia production. Although the fecal VOC profile in cats following OBG supplementation has not yet been reported, previous research has shown that OBG can increase the abundance of cellulolytic bacteria and decrease proteolytic bacteria in the feline gut microbiome [41] and can alter key fecal metabolites, such as indole and *p*-cresol [42]. Collectively, these results suggest that FOS and OBG may contribute to favorable shifts in fecal odor profiles.

However, only three donor cats were included in the present study, and fecal samples were pooled prior to fermentation to obtain a composite inoculum. While pooling reduces the influence of extreme individual variability and facilitates controlled comparison of treatments, it also prevents evaluation of donor-specific responses. Inter-individual variation in the feline gut microbiome is known to be substantial and may influence fermentation patterns and metabolite production [33]. Previous studies have demonstrated that responses to dietary fibers can differ among individuals, particularly when complex or multi-component substrates are used. In contrast, more purified substrates such as FOS often elicit more consistent metabolic responses across individuals, possibly due to functional redundancy within the gut microbiota [43,44]. Therefore, the potential influence of inter-individual microbiome variability should be considered when interpreting differential efficacy observed with complex extracts such as YSE and QSE compared with more defined supplements such as FOS and OBG. Nevertheless, pooling was intentionally applied in this screening-oriented study to provide a standardized microbial background for comparative evaluation of treatments. Future studies incorporating a larger number of individual donors without pooling would be valuable to better characterize variability in response and enhance translational relevance.

This study has some limitations that should be considered when interpreting the findings. The in vitro batch fecal inoculation system may not fully replicate the complexity of in vivo gut fermentation, particularly due to the absence of host physiological influences [45] and the inability to remove end-fermentation products during incubation [46]. For example, modulation of the gut microbiome by host physiological and immune factors plays an important role in shaping fermentation end-products and odor profiles [47,48], and these interactions cannot be captured in an isolated system. These limitations may affect the accuracy of fecal odor estimation compared with in vivo conditions.

Moreover, human sensory evaluation was also not performed, leaving uncertainty regarding the extent to which eNose signal patterns correspond to human olfactory perception. Because odor perception integrates complex neural and cognitive processes [49], validation by trained sensory panels remains essential. Therefore, future studies should incorporate live animal feeding trials and sensory assessments to confirm the practical relevance of these in vitro findings. Despite these constraints, in vitro approaches remain valuable tools for preliminary screening, especially when animal numbers or ethical considerations limit in vivo experimentation. By minimizing host-related confounding factors, in vitro systems facilitate clearer identification of treatment-specific responses and yield more reproducible comparative data [45].

Taken together, these findings suggest that the combination of in vitro batch fecal inoculation and eNose analysis may serve as a practical and efficient screening platform for evaluating dietary deodorant ingredients, while also supporting the odor-modulating effects of prebiotic supplements such as FOS and OBG. These insights provide a useful foundation for future in vivo validation and support the continued development of evidence-based dietary strategies for the control of fecal odor in cats.

## 5. Conclusions

The eNose may serve as a practical and efficient alternative to GC-MS for screening fecal odor profiles in cats. Combining the in vitro batch fecal inoculation system with eNose analysis offered a time- and cost-effective platform for evaluating. Among the tested supplements, the prebiotics FOS and OBG showed superior odor-modulating effects, YSE showed moderate odor-modulating effects, while QSE did not demonstrate meaningful effects. While these highlight the promise of prebiotic ingredients for fecal odor control, further in vivo studies are required to validate their physiological relevance and confirm their applicability under practical feeding conditions.

## Figures and Tables

**Table 1:** Proximate analysis of diet and dry undigested fraction used in the pre-reduced anaerobically sterilized (PRAS) medium (mean ± SD).

Composition	Diet	Dry Undigested Food
Dry matter (% as fed)	95.02 ± 0.01	93.01 ± 0.11
Crude protein (%DM)	35.12 ± 0.11	17.96 ± 0.18
Ether extract (%DM)	9.21 ± 0.02	5.73 ± 0.03
Crude fiber (%DM)	3.68 ± 0.44	13.63 ± 0.40
Crude ash (%DM)	8.36 ± 0.08	24.39 ± 0.51

**Table 2:** Composition of the pre-reduced anaerobically sterilized (PRAS) medium used for in vitro fecal fermentation.

Items	Concentration (g/L)
Dry undigested food	20.0
Cysteine HCl	5.0
KH_2_PO_4_	0.5
K_2_HPO_4_	0.5
NaHCO_3_	5.0
NaCl	1.0
CaCl_2_ 2H_2_O	0.1324
MgSO_4_ 7H_2_O	0.1
Resazurin	0.001

**Table 3:** Mean volatile organic compounds detected by SPME GC-MS at each dilution of fecal samples from ten healthy cats (mean ± SD).

Dilution	Peak Area (Mean ± SD)			
	1:1	1:2	1:4	1:8
Volatile compounds				
[(4-Hexylbenzene-1,3-diyl)bis(oxy)]bis(trimethylsilane)	13,946,790 ± 8,296,799	12,221,813 ± 8,682,135	7,029,629 ± 4,660,518	7,182,835 ± 4,756,178
1H-Indole, 6-methyl	123,394,405 ± 137,766,283	159,226,481 ± 145,738,411	65,547,819 ± 71,500,106	57,370,416 ± 76,272,822
2,5-Cyclohexadiene-1,4-dione,2,6-bis(1,1-dimethylethyl)	3,390,615 ± 2,069,211	3,400,054 ± 1,677,018	1,873,238 ± 857,409	1,160,735 ± 663,999
Acetic acid	84,312,091 ± 36,445,121	33,214,449 ± 25,130,245	10,099,530 ± 5,868,705	9,958,078 ± 10,507,814
Benzaldehyde	36,661,325 ± 19,367,860	18,505,904 ± 8,126,676	7,511,516 ± 3,172,362	3,159,421 ± 999,097
Butanoic acid	455,034,355 ± 395,895,016	100,189,303 ± 128,008,885	37,888,579 ± 65,088,681	11,594,526 ± 12,953,121
Butylated hydroxytoluene	4,176,055 ± 2,126,933	4,922,474 ± 2,048,124	3,025,686 ± 1,550,174	2,008,594 ± 850,692
*p*-Cresol	531,291,360 ± 178,469,283	393,753,060 ± 186,835,091	166,731,933 ± 78,584,161	58,598,515 ± 22,448,472
Cyclotetrasiloxane, octamethyl	22,903,714 ± 7,242,790	27,539,736 ± 24,910,615	12,425,394 ± 2,669,907	10,196,118 ± 3,869,511
Dodecane	3,319,709 ± 1,499,124	967,963 ± 225,857	729,470 ± 244,167	474,715 ± 84,873
Hexadecane	1,438,459 ± 2,859,216	1,746,618 ± 1,871,879	1,113,772 ± 352,206	615,051 ± 194,496
Hexanoic acid	280,949,186 ± 232,813,213	114,054,614 ± 107,204,030	71,798,668 ± 64,674,053	45,892,508 ± 14,512,485
Indole	36,335,677 ± 56,557,365	32,543,231 ± 46,051,777	15,489,112 ± 24,451,525	13,604,119 ± 22,552,838
Indole, 3-methyl	13,354,808 ± 22,826,419	5,845,979 ± 4,543,776	8,679,423 ± 8,917,974	32,820,989 ± 37,553,864
Oxime-, methoxy-phenyl	35,376,925 ± 11,187,166	13,931,137 ± 12,206,519	11,257,880 ± 11,219,549	15,816,037 ± 30,820,730
Pentanoic acid	520,833,153 ± 482,508,570	165,929,444 ± 147,825,974	55,646,725 ± 73,277,199	17,439,047 ± 34,640,083
Phenol	22,189,942 ± 11,971,005	12,774,870 ± 6,398,487	8,093,561 ± 3,917,783	4,764,339 ± 5,332,977
Phenol, 4-ethyl	12,220,595 ± 15,015,654	10,467,609 ± 14,633,951	4,524,924 ± 5,188,126	1,566,699 ± 1,867,831
Propanoic acid	36,102,409 ± 37,721,877	21,945,195 ± 12,012,359	9,927,257 ± 5,936,320	-
Silanediol, dimethyl	36,303,768 ± 7,905,189	32,128,459 ± 8,465,714	30,285,182 ± 12,226,899	24,799,728 ± 12,364,614
Tetradecane	3,840,692 ± 2,232,163	1,422,806 ± 1,173,286	1,016,070 ± 697,880	704,645 ± 310,317

**Table 4:** Mean eNose sensor responses at each dilution of fecal samples from ten healthy cats (mean ± SD).

Dilution	Sensor Response (%) ^1^			
	1:1	1:2	1:4	1:8
Sensor 1	79.83 ± 5.18	77.92 ± 5.59	78.49 ± 1.56	76.62 ± 1.22
Sensor 2	93.61 ± 1.90	91.63 ± 2.73	92.37 ± 1.65	84.46 ± 3.52
Sensor 3	88.43 ± 4.70	80.21 ± 7.93	70.32 ± 4.77	56.47 ± 5.28
Sensor 4	51.32 ± 3.95	44.51 ± 4.36	14.94 ± 2.71	7.91 ± 1.69
Sensor 5	85.98 ± 3.79	75.66 ± 7.20	70.38 ± 3.66	58.39 ± 6.90
Sensor 6	89.22 ± 2.66	85.70 ± 4.35	84.06 ± 1.96	77.68 ± 2.55
Sensor 7	94.92 ± 1.57	93.21 ± 2.29	93.92 ± 1.31	87.61 ± 3.08
Sensor 8	62.65 ± 9.34	42.56 ± 16.66	36.03 ± 6.17	27.54 ± 7.25
Total responses	645.96 ± 28.04	591.40 ± 47.85	540.53 ± 19.49	476.68 ± 26.59

^1^ Descriptions of individual eNose sensors are provided in Supplementary Table S1.

**Table 5:** eNose sensor responses of final in vitro fermentation products with different dietary fecal deodorant supplements at various concentrations.

Treatment ^1,2^	CON	YSE			QSE			FOS			OBG			SEM	p-Value ^3^		
Conc. (g/100 mL)	0.0	0.2	0.4	0.8	0.2	0.4	0.8	0.2	0.4	0.8	0.2	0.4	0.8		T	C	T × C
Sensor 1	85.29 ^a^	84.53 ^b^	83.85 ^b^	84.53 ^b^	83.16 ^b^	81.98 ^b^	83.13 ^b^	82.75 ^b^	83.43 ^b^	84.08 ^b^	83.26 ^b^	83.24 ^b^	81.18 ^b^	0.64	0.21	0.01	0.60
Sensor 2	86.86 ^c^	87.36 ^Bbc^	88.78 ^Bab^	88.81 ^Ba^	82.62 ^Cbc^	83.04 ^Cab^	85.12 ^Ca^	89.89 ^Abc^	92.50 ^Aab^	94.50 ^Aa^	89.40 ^Abc^	91.87 ^Aab^	92.68 ^Aa^	0.67	0.00	0.00	0.00
Sensor 3	79.09 ^ab^	77.93 ^BCb^	79.66 ^BCab^	80.17 ^BCa^	76.37 ^Cb^	76.75 ^Cab^	77.14 ^Ca^	79.20 ^Ab^	83.51 ^Aab^	87.71 ^Aa^	80.32 ^ABb^	82.51 ^ABab^	81.77 ^ABa^	1.02	0.00	0.02	0.10
Sensor 4	59.22 ^a^	58.63 ^ABa^	58.44 ^ABab^	59.34 ^ABb^	60.19 ^Aa^	58.75 ^Aab^	60.84 ^Ab^	56.25 ^Ca^	54.72 ^Cab^	52.06 ^Cb^	59.07 ^BCa^	57.89 ^BCab^	53.44 ^BCb^	0.67	0.00	0.00	0.01
Sensor 5	74.18	76.96	72.86	71.55	74.59	81.02	74.85	75.11	73.05	72.95	78.43	74.20	69.69	1.51	0.38	0.09	0.31
Sensor 6	90.75 ^a^	90.67 ^ab^	89.95 ^b^	90.38 ^b^	89.55 ^ab^	88.11 ^b^	90.07 ^b^	89.37 ^ab^	89.47 ^b^	89.70 ^b^	89.88 ^ab^	90.05 ^b^	87.81 ^b^	0.38	0.14	0.01	0.08
Sensor 7	90.79 ^c^	91.18 ^Bbc^	91.92 ^Bab^	92.05 ^Ba^	87.83 ^Cbc^	88.11 ^Cab^	89.51 ^Ca^	92.64 ^Abc^	94.44 ^Aab^	95.80 ^Aa^	92.29 ^Abc^	93.99 ^Aab^	94.39 ^Aa^	0.44	0.00	0.00	0.00
Sensor 8	76.72 ^a^	78.38 ^Aa^	75.43 ^Aa^	74.90 ^Ab^	76.67 ^Aa^	81.02 ^Aa^	77.24 ^Ab^	73.62 ^Ba^	69.40 ^Ba^	61.91 ^Bb^	79.16 ^Ba^	73.23 ^Ba^	61.44 ^Bb^	0.90	0.00	0.00	0.00
Total *	642.50 ^a^	645.50 ^a^	641.00 ^a^	642.00 ^b^	631.00 ^a^	639.00 ^a^	638.00 ^b^	638.50 ^a^	640.50 ^a^	638.50 ^b^	651.50 ^a^	647.00 ^a^	622.50 ^b^	2.55	0.28	0.04	0.01

^1^ CON, control; YSE, *Yucca shidigera* extract; QSE, *Quilaja saponaria* extract; FOS, fructo oligosaccharide; OBG, beta-glucan. ^2^ Descriptions of individual eNose sensors are provided in Supplementary Table S1. ^3^ T, treatment effect; C, concentration effect; T × C, interaction between treatment and concentration. Superscript letters a–c indicate significant differences (*p* < 0.05) among concentrations according to Tukey’s HSD test. Superscript letters A–C indicate significant differences (*p* < 0.05) among treatments according to Tukey’s HSD test. * For total responses, differences among concentrations were determined using Fisher’s LSD multiple comparison post hoc test.

## Data Availability

The original contributions presented in this study are included in the article/Supplementary Materials. Further inquiries can be directed to the corresponding author.

## Supplementary Materials

The following supporting information can be downloaded at: https://www.mdpi.com/article/10.3390/ani16050801/s1, Table S1: List of eNose sensors and target gases, Table S2: Reference baseline sensor resistance of the eNose sensor array between two measurement days, Figure S1: Line graph showing the reference baseline sensor resistance of the eNose sensor array between two measurement days, Figure S2: Representative sensor (S1) response profile over time between two measurement days.

## Abbreviations

The following abbreviations are used in this manuscript:

DFD	Dietary fecal deodorant
SPME	Solid-phase microextraction
eNose	Electronic nose
YSE	*Yucca schidigera* extract
QSE	*Quillaja saponaria* extract
FOS	Fructooligosaccharides
OBG	Oat beta-glucans
VFA	Volatile fatty acid
BCVFA	Branched-chain volatile fatty acids
VOCs	Volatile organic compounds
PRAS	Pre-reduced anaerobe sterilized
PCA	Principal component analysis
HCA	Hierarchical cluster analysis
