# Supplementary material for: Evaluation of an Electronic Nose Coupled with In Vitro Fecal Fermentation as a Screening Tool for Fecal Odor in Cats
Source: Animals (Basel). 2026 Mar 4;16(5):801. doi: 10.3390/ani16050801 (PMC12984913; doi:10.3390/ani16050801)

Table S1. List of eNose sensors array and target gases.

Sensor	Target gas
S1	Combustible gases
S2	Air pollutants (Hydrogen, Ethanol etc.)
S3	Organic Solvent Vapor
S4	Air pollutants (VOCs, Ammonia, H_2S)
S5	Ammonia
S6	Butane, Propane
S7	Alcohol, Solvent vapors
S8	Ammonia

Table S2. Reference baseline sensor resistance of the eNose sensor array between two measurement days.

	Day 1	Day 2	Mean	SD	%RSD
S1	133969.47	131158.18	132563.82	1405.64	1.06%
S2	115752.01	160798.04	138275.03	22523.02	16.29%
S3	228777.67	226107.37	227442.52	1335.15	0.59%
S4	274949.18	289389.44	282169.31	7220.13	2.56%
S5	79530.26	88228.98	83879.62	4349.36	5.19%
S6	100205.14	108307.37	104256.26	4051.11	3.89%
S7	119620.76	166648.91	143134.83	23514.07	16.43%
S8	50339.24	55094.36	52716.80	2377.56	4.51%

Figure S1. Line graph showing the reference baseline sensor resistance of the eNose sensor array between two measurement days

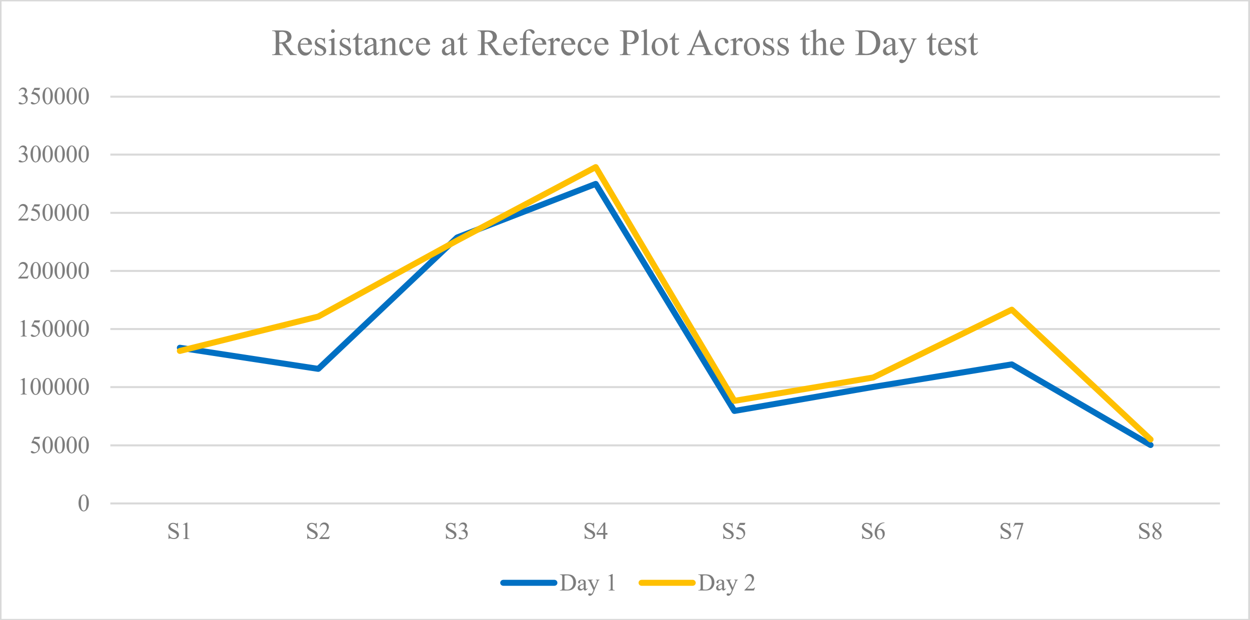

Figure S2. Representative response profile of sensor S1 over time across two measurement days.

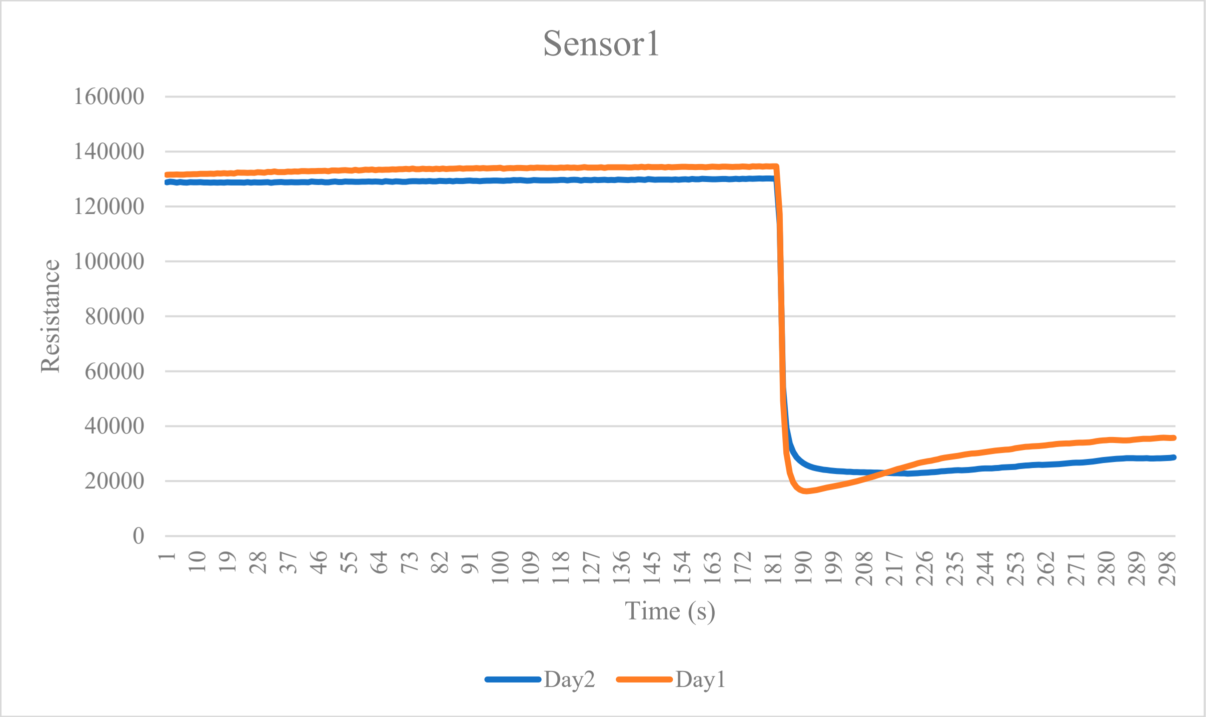

Supplement: Supplementary file 1 [file animals-16-00801-s001.zip › animals-4119257-supplementary.pdf]
